# Biomarkers for primary graft dysfunction after lung transplantation: a review of current evidence and future prospects

**DOI:** 10.3389/fphys.2025.1557182

**Published:** 2025-05-22

**Authors:** Li Feng, Kelin Luo, Yu Qiu, Rui Li, Chun Xue, Shuaishuai Xi, Jixian Liu, Yuanmin Pei, Chao Ma

**Affiliations:** ^1^ School of Clinical Medicine, Shandong Second Medical University, Weifang, Shandong, China; ^2^ Department of Gynecology and Obstetrics, The Second Affiliated Hospital of Naval Medical University (Shanghai Changzheng Hospital), Shanghai, China; ^3^ Department of Thyroid and Breast Surgery, Jinan Third People’s Hospital, Jinan, Shandong, China; ^4^ Department of Vascular Surgery, Yidu Central Hospital of Weifang, Weifang, Shandong, China; ^5^ Department of Thoracic Surgery, Peking University Shenzhen Hospital, Shenzhen, Guangdong, China

**Keywords:** primary graft dysfunction, lung transplantation, biomarker, plasma proteins, cell-free DNA

## Abstract

Lung transplantation remains the only effective treatment for end-stage lung disease, offering the potential to significantly prolong survival and enhance quality of life for recipients. However, primary graft dysfunction (PGD)-a severe form of lung injury occurring within the first 72 h post-transplantation-constitutes a major cause of early mortality and presents a substantial barrier to the broader clinical adoption of lung transplantation. Biomarkers, defined as specific molecules, cells, or other biological indicators detectable within or outside the body, can reflect physiological states, disease progression, or therapeutic responses. The identification of accurate and reliable biomarkers for the prediction and diagnosis of PGD is therefore critical for improving diagnostic precision and therapeutic outcomes. This review provides a comprehensive overview of recent advances in the discovery of PGD-related biomarkers, encompassing a wide range of candidates such as plasma proteins, hormones, cell-free DNA, and immunoreactive substances. The complex biomarker landscape associated with PGD involves multiple signaling pathways and cellular phenotypes. Despite ongoing research, no single biomarker has yet demonstrated sufficient predictive or diagnostic power to be used independently in clinical practice. Consequently, continued investigation is essential to validate existing biomarkers and develop optimized strategies for their integration into routine clinical application.

## Introduction

Lung transplantation remains an effective treatment for end-stage lung diseases, although its success is often limited by primary graft dysfunction (PGD) (Shah and Diamond, 2018; Young and Dilling, 2019; Avtaar Singh et al., 2023). PGD presents as a severe form of lung injury within the first 72 h after transplantation and is characterized by hypoxemia, pulmonary edema, and decreased pulmonary compliance (Criner et al., 2021). With an incidence rate as high as 30%–50%, PGD is strongly associated with both early and long-term post-transplant mortality (Hunt and Cantu, 2023). Currently, there is a lack of reliable biomarkers and pharmacological treatments for PGD, making early diagnosis and intervention challenging. Therefore, identifying biomarkers that can predict, diagnose, and potentially guide treatment for PGD is a pressing need in the field of lung transplantation.

Biomarkers are substances that reflect physiological or pathological states and can be detected in bodily fluids such as blood, urine, bronchoalveolar lavage fluid (BALF), or organ perfusate to evaluate organ function or injury (Calfee and Ware, 2007; Chacon-Alberty et al., 2022; Hamilton et al., 2017; Nakata et al., 2023). In recent years, advances in molecular biology, proteomics, and metabolomics have led to some progress in identifying potential PGD biomarkers. However, challenges remain, including small sample sizes, inconsistent findings, and a lack of thorough validation.

This review aims to summarize current research on PGD biomarkers, including plasma proteins, hormones, cell-free DNA, and immunoreactive substances. It discusses their advantages, limitations, and potential clinical applications, while also highlighting the challenges and future directions in biomarker discovery for PGD.

## Primary graft dysfunction and biomarkers

**FIGURE 1:**
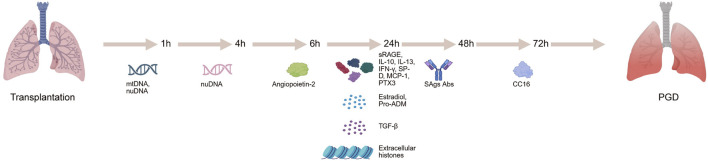
Identification of biomarkers in primary graft dysfunction can be evaluated at different time points in lung transplantation recipients.

Biomarkers are substances that reflect physiological or pathological states, including genes, proteins, metabolites, and cytokines (Lozano-Edo et al., 2022; Lee and Christie, 2011; Suzuki et al., 2013). They can be detected in bodily fluids such as blood, urine, BALF, or organ perfusate, and are used to assess organ function or the extent of injury. PGD is one of the most common complications following lung transplantation, significantly affecting patient survival and quality of life (Wu et al., 2023). As such, identifying reliable biomarkers for the early prediction and accurate diagnosis of PGD remains a critical goal in the field. Despite encouraging progress, most studies on PGD biomarkers have been limited by relatively small sample sizes, and further research is needed to validate these findings for clinical use. Currently, PGD diagnosis primarily depends on the oxygenation index, chest radiographs, and clinical judgment (Sanchez-Gonzalez et al., 2022; Li et al., 2021; Neves et al., 2016). However, these methods often lack sensitivity and specificity, and may not effectively capture the onset or resolution of PGD. The discovery of biomarkers capable of predicting PGD risk, monitoring its progression, and guiding treatment strategies holds substantial clinical promise (Figure 1). Advancing biomarker research could ultimately transform the diagnosis, management, and outcomes of PGD in lung transplant recipients. Table 1 summarizes the detailed information of biomarkers for PGD following lung transplantation.

**TABLE 1:** Overview of biomarkers for primary graft dysfunction after lung transplantation.

Biomarker	Biological fucntion	Number of patients	Single/multi-center	Time points of biomarker detection after lung transplantation (/h)	With PGD	Without PGD	Detection method	References
Plasma Proteins								
sRAGE	Soluble receptor	317	multi-center	24	4.3 ng/mL	1.9 ng/mL	Sandwich ELISA	Christie et al. (2009)
IL-10	Anti-inflammatory cytokine	80	single-center	24	3(PGD1)/5.5(PGD2)/10.9(PGD3) pg/mL	2 pg/mL	ELISA	Frick et al. (2020)
IL-13	Inflammatory cytokine	80	single-center	24	0.2(PGD1)/1(PGD2)/0.9(PGD3) pg/mL	0.7 pg/mL	ELISA	Frick et al. (2020)
IFN-γ	Immune response regulator	80	single-center	24	5.7(PGD1)/8.4(PGD2)/11.5(PGD3) pg/mL	4.8 pg/mL	ELISA	Frick et al. (2020)
SP-D	Pulmonary surfactant	80	single-center	24	3841(PGD1)/4247(PGD2)/3263(PGD3) pg/mL	6001 pg/mL	ELISA	Frick et al. (2020)
CC16	Secretory protein	104	multi-center	72	13.8 ng/mL	8.2 ng/mL	Sandwich ELISA	Diamond et al. (2011a)
MCP-1	Chemotactic factor	108	multi-center	24	167.95 pg/mL	103.5 pg/mL	ELISA	Shah et al. (2012)
Angiopoietin-2	Vascular growth stimulator	119	multi-center	6	4578 pg/mL	3218 pg/mL	ELISA	Diamond et al. (2017)
VEGF	Angiogenesis factor	150	single-center	72	584(PGD1)/829(PGD2)/1191(PGD3)/pg/mL	428 pg/mL	ELISA	Krenn et al. (2007)
PTX3	Complement system regulator	119	multi-center	24	88.9 ng/mL	22.7 ng/mL	Sandwich ELISA	Diamond et al. (2011b)
Hormones								
Estradiol	Estrogen	111	multi-center	24	77.4 pg/mL	59.6 pg/mL	ELISA	Bastarache et al. (2012)
Pro-ADM	Precursor of adrenomedullin	100	single-center	24	3.25 nmol/L	1.61 nmol/L	An immune time-resolved amplified cryptate emission technology assay	Riera et al. (2016)
Cell-free DNA								
cfDNA	Fragmented DNA	186	multi-center	NA	23.7 ng/mL	12.9 ng/mL	Quantitative PCR	Balasubramanian et al. (2024)
mtDNA	Mitochondrial DNA	62	single-center	1	1874 copies/μL	1259 copies/μL	Quantitative PCR	Kanou et al. (2021)
nuDNA	Nuclear DNA	62	single-center	1	1895 copies/μL	675 copies/μL	Quantitative PCR	Kanou et al. (2021)
nuDNA	Nuclear DNA	62	single-center	4	4521 copies/μL	1764 copies/μL	Quantitative PCR	Kanou et al. (2021)
%ddcfDNA	Percentage donor-derived cell-free DNA	99	multi-center	72	12.2%	8.5%	An automated shotgun sequencing method	Keller et al. (2021)
Immunoreactive Substances								
TGF-β	Cytokine	279	single-center	24	-	-	ELISA	DerHovanessian et al. (2016)
SAgs Abs	lung-associated self-antigen antibodies	317	multi-center	48	-	-	ELISA	Tiriveedhi et al. (2013)
Others								
Extracellular histones	Cytotoxic proteins	65	single-center	24	5.882 μg/ml	2.478 μg/ml	Sandwich ELISA	Jin et al. (2020)

## Plasma proteins

Plasma proteins are essential for maintaining homeostasis, facilitating substance transport, supporting immune defense, and regulating coagulation (Christie et al., 2009; Covarrubias et al., 2007; Leon et al., 2009). A retrospective analysis examined the relationship between plasma cytokine levels before and after lung transplantation and the severity of PGD (Frick et al., 2020). Of the 30 proteins tested, eight showed significant differences between patients with mild and severe PGD: IL-6, IL-10, IL-13, eotaxin, G-CSF, IFN-γ, MIP-1α, and SP-D. Notably, IL-10 and IL-13 were associated with prolonged extubation times, extended ICU stays, and longer overall hospitalizations, independent of donor and recipient characteristics. Plasma IL-10 and IFN-γ levels in both donors and recipients correlated positively with PGD incidence and severity, whereas SP-D levels were inversely correlated with PGD severity.

Patients with PGD typically exhibit elevated levels of inflammatory mediators in early post-transplant serum, such as MCP-1, IP-10, IL-1β, IL-2, IFN-γ, and IL-12 (21). Further investigations revealed that PGD-induced inflammation may enhance donor HLA class II antigen expression on the graft, increase antigen presentation, and stimulate donor-specific immune responses (Bharat et al., 2008).

In a multicenter cohort study, protein C and plasminogen activator inhibitor-1 (PAI-1) levels were measured in the plasma of lung transplant recipients (Christie et al., 2007). Those who developed PGD had significantly lower post-transplant protein C levels and higher PAI-1 levels compared to those without PGD. Pre-transplant pulmonary artery systolic pressure was positively correlated with post-transplant PAI-1 levels, potentially linking pulmonary hypertension to PGD development.

Another study collected blood samples from lung transplant recipients before surgery and at 6, 24, 48, and 72 h post-transplant to evaluate plasma ICAM-1 and von Willebrand factor (vWF) levels (Covarrubias et al., 2007). ICAM-1 is a cell adhesion molecule predominantly expressed on the surface of endothelial and immune cells (Singh et al., 2023; Bui et al., 2020; van de Stolpe and van der Saag, 1996). It plays a critical role in the inflammatory response by interacting with integrins on leukocytes, thereby facilitating their adhesion and migration across the endothelium. This process promotes the infiltration of inflammatory cells into injured tissues. vWF is a large glycoprotein synthesized and secreted by endothelial cells (Zanetta et al., 2000; Xiang and Hwa, 2016; Nakhaei-Nejad et al., 2019). It is primarily involved in hemostasis, contributing to blood coagulation and platelet aggregation. ICAM-1 levels were significantly higher in patients with PGD and positively correlated with PGD occurrence. Although vWF levels tended to rise postoperatively, they were not significantly associated with PGD. ICAM-1 levels also correlated with pre-transplant pulmonary artery pressure and recipient diagnosis (Covarrubias et al., 2007).

CC16, a protein secreted by airway epithelial cells, has been significantly associated with PGD, particularly in recipients without idiopathic pulmonary fibrosis (non-IPF) (Diamond et al., 2011a). Produced by non-ciliated pulmonary epithelial cells, CC16 may serve as a biomarker for epithelial injury (Shah et al., 2014). In a prospective cohort, CC16 levels at 6 h post-transplant were notably higher in PGD patients, and a 15 ng/mL increase in CC16 was linked to a 1.6-fold higher PGD risk. Additionally, MCP-1, a chemotactic protein released by pulmonary epithelial cells, plays a key role in recruiting inflammatory cells and mediating ischemia-reperfusion injury (IRI) (Yoshimura, 2018; Singh et al., 2021; Ferreira et al., 2005). Elevated MCP-1 levels at 24 h post-transplant were positively correlated with PGD risk and severity within 72 h (Shah et al., 2012).

Angiopoietin-2 (Ang2), a vascular growth stimulator involved in angiogenesis, binds to the TIE2 receptor and acts as a negative regulator of the ANG1/TIE2 signaling pathway, modulating endothelial responses to cytokines (Akwii et al., 2019; Scholz et al., 2015; Song et al., 2012; Eklund et al., 2017; Nicolini et al., 2019). In PGD patients, Ang2 plasma levels changed significantly over time, particularly in those with idiopathic pulmonary fibrosis (IPF), but showed no significant association in chronic obstructive pulmonary disease (COPD) patients (Diamond et al., 2017).

Vascular endothelial growth factor (VEGF), a key regulator of vascular permeability, was evaluated preoperatively in lung transplant patients (Guzmán et al., 2023; Shi et al., 2022). VEGF serum levels were significantly higher in patients who developed grade 3 PGD compared to those with lower-grade PGD or healthy controls. Elevated VEGF levels were predictive of more severe PGD outcomes (Krenn et al., 2007).

Long pentraxin-3 (PTX3), a protein involved in complement activation and regulation, has also been linked to PGD (Cieślik and Hrycek, 2012; Chiari et al., 2023). In IPF recipients, PTX3 levels at 6 and 24 h post-transplant correlated positively with PGD risk (Diamond et al., 2011b). Genetic analysis in lung transplant recipients identified two PTX3 gene polymorphisms associated with increased PGD risk (Diamond et al., 2012).

The receptor for advanced glycation end-products (RAGE), a transmembrane protein of the immunoglobulin superfamily, has a soluble form (sRAGE) that includes its extracellular domain (Eva et al., 2022; Yue et al., 2022; Bongarzone et al., 2017). sRAGE levels measured at 6 and 24 h post-transplant were higher in PGD patients. These levels were influenced by right heart pressure and cardiopulmonary bypass and were associated with red blood cell transfusion and bypass usage (Kim T. et al., 2023).

### Hormones

Prostaglandin E2 (PGE2) is a hormone-like lipid compound that plays a key role in numerous physiological processes, including smooth muscle contraction and relaxation, vasodilation and vasoconstriction, blood pressure regulation, and the modulation of inflammation (Képes et al., 2023; Cheng et al., 2021; Finetti et al., 2020; Finetti et al., 2023). In a large-scale gene association study, 17 genetic variants were significantly linked to PGD, four of which were located within genes related to the PGE2 pathway (Diamond et al., 2014). One notable variant involved a coding change in the PTGES2 gene, resulting in the substitution of arginine with histidine at position 298, which was associated with an increased risk of PGD. The other three variants were found in the promoter region and first intron of the PTGER4 gene and were associated with a decreased risk of PGD. Functional analysis showed that the rs4434423A variant in PTGER4 influenced the inhibitory function of regulatory T cells.

In another study, plasma estradiol levels were measured before transplantation and at 6 and 24 h post-transplantation to assess their relationship with PGD severity within 72 h after surgery. While no significant differences were found between male and female recipients overall, a positive correlation between estradiol levels at 24 h and PGD severity was observed in male recipients. This association was not present in female recipients (Bastarache et al., 2012).

Pro-adrenomedullin (pro-ADM), a precursor of adrenomedullin (ADM), has been identified as a potential biomarker in various acute conditions such as sepsis, acute heart failure, cardiac arrest, and stroke (Liang et al., 2023; Hagag et al., 2011; Spoto et al., 2023; Zelniker et al., 2023; Ishiyama et al., 2023). It reflects the rapid breakdown of ADM in circulation. In a prospective study of lung transplant recipients, pro-ADM levels were measured at 24, 48, and 72 h following ICU admission (Riera et al., 2016). Findings indicated that pro-ADM levels were strongly correlated with PGD severity and positively associated with ICU mortality. Patients with PGD grade 3 exhibited significantly higher pro-ADM levels at 72 h. Furthermore, pro-ADM levels measured at 24 h could predict the development of PGD grade 3 by 72 h. The predictive value of pro-ADM for ICU mortality surpassed that of PGD grading alone, and combining both enhanced prognostic accuracy. Elevated pro-ADM levels were strongly linked to early graft dysfunction and post-transplant mortality.

Endothelin-1 (ET-1) is a potent peptide hormone known for its vasoconstrictive and proliferative effects and plays a critical role in the pathogenesis of various pulmonary diseases (Banecki and Dora, 2023; Salama et al., 2010). In a study analyzing lung tissue and serum samples from lung transplant donors and recipients, both ET-1 mRNA expression in lung tissue and serum ET-1 concentrations were positively correlated with the severity of PGD(65).

Follistatin-like protein 1 (FSTL1) is a secretory glycoprotein involved in multiple biological functions, including the regulation of myocardial ischemia-reperfusion injury, airway remodeling, and inflammatory responses (Veraar et al., 2022; Kim DK. et al., 2023; Chiou et al., 2019). In a prospective cohort of bilateral lung transplant recipients with end-stage lung disease, post-transplant elevations in plasma FSTL1 levels were identified, showing significant associations with the incidence and clinical severity of PGD(66).

### Cell-free DNA

Cell-free DNA (cfDNA) refers to the free DNA fragments existing in the extracellular environment (Valpione et al., 2018; Mattox et al., 2023). It mainly originates from apoptosis, necrosis, inflammatory responses, and tumor cells. Present in bodily fluids such as blood, saliva, and urine in the form of short fragments, cfDNA is characterized by its diverse sources, fragmentation, and short half-life (Bruhm et al., 2025; Hu et al., 2021; Sherwood and Weimer, 2018). Under normal physiological conditions, the concentration of cfDNA in the bloodstream of healthy individuals remains relatively low. However, in conditions that accelerate cell turnover, such as acute or chronic inflammation, cfDNA levels can rise significantly. Given its short half-life-typically less than one to two hours-cfDNA can serve as a real-time biomarker for disease. By distinguishing between donor- and recipient-specific single nucleotide polymorphisms (SNPs), the origin of circulating cfDNA can be determined, enabling the detection of donor-derived cfDNA. This has potential applications in identifying graft injury following transplantation (Magnusson et al., 2022; Li and Liang, 2023; Zou et al., 2017; Keller and Agbor-Enoh, 2021; Balasubramanian et al., 2024; Ju et al., 2023; Keller et al., 2022a; Keller et al., 2024; Kim et al., 2024; Keller et al., 2022b; Zhang et al., 2024; Keller MB. et al., 2022).

In lung transplant recipients, cfDNA levels are almost twice as high as those observed in healthy individuals, primarily originating from innate immune cells involved in inflammatory responses (Keller and Agbor-Enoh, 2022). Elevated cfDNA levels prior to transplantation are associated with a heightened risk of post-transplant pulmonary edema (such as PGD) and mortality. These levels also show potential in predicting both early and long-term complications, such as PGD, chronic lung allograft dysfunction (CLAD), and death, making cfDNA a promising molecular tool for risk stratification in transplant recipients (Keller et al., 2024; Keller and Agbor-Enoh, 2022).

Analysis of perfusate from donor lungs at one and 4 hours of perfusion revealed significantly higher levels of cfDNA-including mitochondrial DNA (mtDNA) and nuclear DNA (nuDNA)-in lungs that later developed severe PGD (grade 3) within 72 h after transplantation, particularly those from donation after circulatory death (DCD) donors. While cfDNA shows promise as a predictive marker for PGD, its diagnostic accuracy still requires further refinement (Kanou et al., 2021).

In a prospective study involving lung transplant recipients, plasma samples collected on days 1, 3, and 7 post-transplantation revealed that patients who developed PGD had elevated levels of percentage donor-derived cell-free DNA (%ddcfDNA). These levels correlated positively with the severity of PGD. Furthermore, PGD patients with higher %ddcfDNA levels were at increased risk for developing CLAD. Notably, %ddcfDNA levels in PGD patients who progressed to CLAD were approximately double those in PGD patients who did not develop CLAD (Keller et al., 2021).

### Immunoreactive substances

Surfactant protein A (SP-A) is a key pulmonary surfactant involved in immune defense and the regulation of inflammation in the lungs (D'Ovidio et al., 2013; Depicolzuane et al., 2021; King and Chen, 2020). Low expression of SP-A mRNA in donor lungs has been significantly associated with reduced post-transplant survival. After transplantation, recipients with low SP-A mRNA levels show decreased SP-A concentrations in BALF, elevated levels of IL-2 and IL-12, and an increased incidence of rejection episodes (D'Ovidio et al., 2013).

Elevated levels of CCR5 and its ligands have been observed in both mouse and human models of ischemia-reperfusion injury (IRI). CCR5-positive natural killer (NK) cells accumulate in the lungs and airways, exhibiting markers of maturity and tissue residency. The CCR5 antagonist maraviroc has been shown to reduce NK cell migration to the airways, decrease pulmonary vascular permeability, improve oxygenation, and lower the incidence and severity of PGD (Santos et al., 2023).

Dectin-1, a C-type lectin receptor, plays a role in recognizing and activating a variety of ligands, including β-glucans, endogenous damage-associated molecular patterns (DAMPs), and fungal pathogen-associated molecular patterns (PAMPs) (Ochoa et al., 2023; Drummond et al., 2022; Yang et al., 2023). It is involved in modulating inflammatory responses and immune tolerance. A specific Dectin-1 mutation (Y238X) has been linked to acute rejection after lung transplantation, increased lymphocyte proportions in BALF, the development of bronchiolitis obliterans syndrome (BOS) (Calabrese et al., 2019). Additionally, levels of transforming growth factor-beta (TGF-β) increase during PGD and are associated with BOS development. Immunohistochemistry has revealed TGF-β expression in epithelial cells, interstitial cells, and macrophages in transplanted lungs, suggesting that TGF-β may serve as a critical mediator linking PGD and BOS, and could potentially function as a biomarker for both conditions (DerHovanessian et al., 2016).

In a large cohort of lung transplant recipients, patients were grouped by underlying conditions-such as COPD, IPF, and cystic fibrosis (CF)-to investigate the relationship between antibodies against lung-associated self-antigens (SAgs) and PGD. The highest pre-transplant positivity rates for SAg antibodies were observed in the IPF and CF groups. Recipients with pre-existing SAg antibodies exhibited higher rates of PGD and elevated serum levels of inflammatory cytokines (Tiriveedhi et al., 2013).

Type V collagen (col(V)), primarily located at the apex of lung epithelial cells, has been shown to induce complement-dependent cytotoxicity (Mak et al., 2016; Iwata et al., 2008; Zaffiri et al., 2019). In lung transplant recipients, high pre-transplant plasma levels of anti-col(V) antibodies have been significantly associated with severe PGD following surgery. Lung-restricted autoantibodies (LRAs) are recognized as important pathogenic contributors to PGD, with mechanisms involving IL-1β-mediated increases in pulmonary vascular endothelial permeability and activation of the complement cascade. These findings offer promising targets for preventive and therapeutic strategies (Yang et al., 2022).

Genetic studies have identified a variant (rs3168046) in the Toll-interacting protein (TOLLIP) gene that is significantly associated with PGD, as well as with plasma levels of plasminogen activator inhibitor-1 (PAI-1) (Cantu et al., 2016). Additionally, two IL-17 receptor (IL-17R) gene variants (rs882643 and rs2241049) have been linked to increased risk of PGD, with carriers of the risk genotypes more likely to experience higher PGD grades within the first 48 h post-transplantation (Somers et al., 2015).

### Others

Extracellular histones are a novel class of highly tissue-damaging molecules released during cell death and the formation of neutrophil extracellular traps (NETs). These molecules exhibit diverse biological activities, including cytotoxic effects, promotion of inflammation, and enhancement of platelet aggregation (Jin et al., 2020; de Vries et al., 2023; Zhong et al., 2023). Following lung transplantation, extracellular histone levels increase significantly, particularly in patients who develop PGD. *In vitro* studies have shown that serum collected within 24 h post-transplantation from patients with high extracellular histone levels is markedly toxic to human pulmonary artery endothelial cells (HPAECs) and stimulates cytokine production in human monocytic cell lines (THP1). These effects are largely mitigated by heparin or anti-histone antibodies (Jin et al., 2020).

Telomere length in airway epithelial cells has also been associated with PGD severity (Greenland et al., 2023). In one analysis of lung transplant recipients, a negative correlation was observed between telomere length in airway epithelial cells and the severity of PGD within the first few weeks after transplantation. Further evidence suggests that PGD may contribute to telomere dysfunction, thereby enhancing immune activation. Telomere impairment in airway epithelial cells may represent a mechanistic link between PGD and the later development of CLAD (Spahn et al., 2015).

The transient receptor potential vanilloid 4 (TRPV4) channel in endothelial cells has emerged as a key mediator of lung IRI. Inhibition or genetic deletion of TRPV4 significantly improves pulmonary function, reduces pulmonary edema and inflammatory cell infiltration, and lowers levels of inflammatory cytokines. These findings suggest that TRPV4 channels may serve as promising therapeutic targets for preventing PGD after lung transplantation (Kuppusamy et al., 2023; Weber et al., 2020).

## Limitations and future prospects

Although numerous biomarkers associated with PGD have been identified, most current studies are limited by small sample sizes and a lack of multicenter validation. Therefore, there is an urgent need for further research to identify biomarkers with high sensitivity and specificity, and to develop standardized detection methods and diagnostic criteria.

PGD is a dynamic and evolving condition, with biomarker levels fluctuating over time to reflect different pathophysiological mechanisms and prognostic implications. Thus, determining the optimal timing and frequency for biomarker collection, as well as defining clinically relevant thresholds, is crucial for early prediction and real-time monitoring. However, threshold values for biomarkers vary across studies, which may be due to differences in sample sizes, study designs, detection methods, and patient populations. The biomarkers highlighted in this review offer advantages such as higher sensitivity, stronger specificity, and the ability to guide therapeutic adjustments. For instance, cytokines like IL-10 and IL-13 show significantly elevated levels in early post-transplant serum and are closely associated with PGD severity. These changes often precede the appearance of clinical symptoms, enabling physicians to identify high-risk patients before PGD fully develops. Additionally, certain biomarkers are closely linked to the pathogenesis of PGD and demonstrate high specificity. For example, SP-A, which plays a key role in pulmonary immune defense and inflammation regulation, has been shown to correlate with reduced post-transplant survival when expressed at low levels in donor lungs. Such biomarkers, directly involved in the pathogenesis of PGD, more accurately reflect post-transplant pathological states and reduce the risk of misdiagnosis.

Furthermore, monitoring biomarker fluctuations allows clinicians to more precisely assess PGD severity and progression, facilitating timely therapeutic adjustments. For example, elevated levels of circulating cfDNA are strongly associated with both the occurrence and severity of PGD, as well as an increased risk of CLAD. By measuring cfDNA levels, physicians can identify patients at risk of developing CLAD in advance and implement appropriate preventive or therapeutic strategies.

Given the complexity and heterogeneity of PGD, a single biomarker may be insufficient for accurate diagnosis or prognosis. Therefore, combining and integrating multiple biomarkers may improve diagnostic precision. Advanced analytical approaches, such as multivariate statistical analyses and machine learning, can support the development of composite scoring systems or predictive models. Currently, most biomarker detection methods rely on ELISA and quantitative PCR, which are cost-effective and easily implemented. However, detection methods for some emerging biomarkers are still under development.

Biomarkers not only serve diagnostic and prognostic roles but may also act as therapeutic targets. Future research should focus on elucidating the functional roles, regulatory mechanisms, and detection strategies of these biomarkers, paving the way for effective prevention and treatment strategies for PGD.

## References

[B1] Akwii R. G. Sajib M. S. Zahra F. T. Mikelis C. M. (2019). Role of angiopoietin-2 in vascular physiology and pathophysiology. Cells 8 (5), 471. 10.3390/cells8050471 31108880 PMC6562915

[B2] Avtaar Singh S. S. Das De S. Al-Adhami A. Singh R. Hopkins P. M. Curry P. A. (2023). Primary graft dysfunction following lung transplantation: from pathogenesis to future frontiers. World J. Transpl. 13 (3), 58–85. 10.5500/wjt.v13.i3.58 PMC10037231 36968136

[B3] Balasubramanian S. Richert M. E. Kong H. Fu S. Jang M. K. Andargie T. E. (2024). Cell-free DNA maps tissue injury and correlates with disease severity in lung transplant candidates. Am. J. Respir. Crit. Care Med. 209 (6), 727–737. Epub 2023/12/20. 10.1164/rccm.202306-1064OC 38117233 PMC10945061

[B4] Banecki KMRM Dora K. A. (2023). Endothelin-1 in Health and disease. Int. J. Mol. Sci. 24 (14), 11295. 10.3390/ijms241411295 37511055 PMC10379484

[B5] Bastarache J. A. Diamond J. M. Kawut S. M. Lederer D. J. Ware L. B. Christie J. D. (2012). Postoperative estradiol levels associate with development of primary graft dysfunction in lung transplantation patients. Gend. Med. 9 (3), 154–165. 10.1016/j.genm.2012.01.009 22361838 PMC3374892

[B6] Bharat A. Kuo E. Steward N. Aloush A. Hachem R. Trulock E. P. (2008). Immunological link between primary graft dysfunction and chronic lung allograft rejection. Ann. Thorac. Surg. 86 (1), 189–195. ; discussion 96-97. 10.1016/j.athoracsur.2008.03.073 18573422 PMC2790810

[B7] Bongarzone S. Savickas V. Luzi F. Gee A. D. (2017). Targeting the receptor for advanced glycation endproducts (rage): a medicinal chemistry perspective. J. Med. Chem. 60 (17), 7213–7232. 10.1021/acs.jmedchem.7b00058 28482155 PMC5601361

[B8] Bruhm D. C. Vulpescu N. A. Foda Z. H. Phallen J. Scharpf R. B. Velculescu V. E. (2025). Genomic and fragmentomic landscapes of cell-free DNA for early cancer detection. Nat. Rev. Cancer 25, 341–358. 10.1038/s41568-025-00795-x 40038442 PMC13003562

[B9] Bui T. M. Wiesolek H. L. Sumagin R. (2020). Icam-1: a master regulator of cellular responses in inflammation, injury resolution, and tumorigenesis. J. Leukoc. Biol. 108 (3), 787–799. Epub 2020/03/18. 10.1002/jlb.2mr0220-549r 32182390 PMC7977775

[B10] Calabrese D. R. Wang P. Chong T. Hoover J. Singer J. P. Torgerson D. (2019). Dectin-1 genetic deficiency predicts chronic lung allograft dysfunction and death. JCI Insight 4 (22), e133083. 10.1172/jci.insight.133083 31613800 PMC6948872

[B11] Calfee C. S. Ware L. B. (2007). Biomarkers of lung injury in primary graft dysfunction following lung transplantation. Biomark. Med. 1 (2), 285–291. 10.2217/17520363.1.2.285 20477403

[B12] Cantu E. Suzuki Y. Diamond J. M. Ellis J. Tiwari J. Beduhn B. (2016). Protein quantitative trait loci analysis identifies genetic variation in the innate immune regulator tollip in post-lung transplant primary graft dysfunction risk. Am. J. Transpl. 16 (3), 833–840. 10.1111/ajt.13525 PMC4767612 26663441

[B13] Chacon-Alberty L. Kanchi R. S. Ye S. Hochman-Mendez C. Daoud D. Coarfa C. (2022). Plasma protein biomarkers for primary graft dysfunction after lung transplantation: a single-center cohort analysis. Sci. Rep. 12 (1), 16137. 10.1038/s41598-022-20085-y 36167867 PMC9515157

[B14] Cheng H. Huang H. Guo Z. Chang Y. Li Z. (2021). Role of prostaglandin E2 in tissue repair and regeneration. Theranostics 11 (18), 8836–8854. 10.7150/thno.63396 34522214 PMC8419039

[B15] Chiari D. Pirali B. Perano V. Leone R. Mantovani A. Bottazzi B. (2023). The crossroad between autoimmune disorder, tissue remodeling and cancer of the thyroid: the long pentraxin 3 (Ptx3). Front. Endocrinol. (Lausanne) 14, 1146017. 10.3389/fendo.2023.1146017 37025408 PMC10070760

[B16] Chiou J. Chang Y.-C. Tsai H.-F. Lin Y.-F. Huang M.-S. Yang C.-J. (2019). Follistatin-like protein 1 inhibits lung cancer metastasis by preventing proteolytic activation of osteopontin. Cancer Res. 79 (24), 6113–6125. 10.1158/0008-5472.CAN-19-0842 31653686

[B17] Christie J. D. Robinson N. Ware L. B. Plotnick M. De Andrade J. Lama V. (2007). Association of protein C and type 1 plasminogen activator inhibitor with primary graft dysfunction. Am. J. Respir. Crit. Care Med. 175 (1), 69–74. 10.1164/rccm.200606-827OC 17023732 PMC1899260

[B18] Christie J. D. Shah C. V. Kawut S. M. Mangalmurti N. Lederer D. J. Sonett J. R. (2009). Plasma levels of receptor for advanced glycation end products, blood transfusion, and risk of primary graft dysfunction. Am. J. Respir. Crit. Care Med. 180 (10), 1010–1015. 10.1164/rccm.200901-0118OC 19661249 PMC2778153

[B19] Cieślik P. Hrycek A. (2012). Long pentraxin 3 (Ptx3) in the light of its structure, mechanism of action and clinical implications. Autoimmunity 45 (2), 119–128. 10.3109/08916934.2011.611549 21988562

[B20] Covarrubias M. Ware L. B. Kawut S. M. De Andrade J. Milstone A. Weinacker A. (2007). Plasma intercellular adhesion molecule-1 and von Willebrand factor in primary graft dysfunction after lung transplantation. Am. J. Transpl. 7 (11), 2573–2578. 10.1111/j.1600-6143.2007.01981.x 17908278

[B21] Criner R. N. Clausen E. Cantu E. (2021). Primary graft dysfunction. Curr. Opin. Organ Transpl. 26 (3), 321–327. 10.1097/MOT.0000000000000876 33938469

[B22] Depicolzuane L. Phelps D. S. Floros J. (2021). Surfactant protein-a function: knowledge gained from sp-a knockout mice. Front. Pediatr. 9, 799693. 10.3389/fped.2021.799693 35071140 PMC8777267

[B23] DerHovanessian A. Weigt S. S. Palchevskiy V. Shino M. Y. Sayah D. M. Gregson A. L. (2016). The role of tgf-Β in the association between primary graft dysfunction and bronchiolitis obliterans syndrome. Am. J. Transpl. 16 (2), 640–649. 10.1111/ajt.13475 PMC4946573 26461171

[B24] de Vries F. Huckriede J. Wichapong K. Reutelingsperger C. Nicolaes G. A. F. (2023). The role of extracellular histones in covid-19. J. Intern Med. 293 (3), 275–292. 10.1111/joim.13585 36382685 PMC10108027

[B25] Diamond J. M. Akimova T. Kazi A. Shah R. J. Cantu E. Feng R. (2014). Genetic variation in the prostaglandin E2 pathway is associated with primary graft dysfunction. Am. J. Respir. Crit. Care Med. 189 (5), 567–575. 10.1164/rccm.201307-1283OC 24467603 PMC3977709

[B26] Diamond J. M. Cantu E. Porteous M. K. Suzuki Y. Meyer K. C. Lederer D. J. (2017). Peripheral blood gene expression changes associated with primary graft dysfunction after lung transplantation. Am. J. Transpl. 17 (7), 1770–1777. 10.1111/ajt.14209 PMC5489369 28117940

[B27] Diamond J. M. Kawut S. M. Lederer D. J. Ahya V. N. Kohl B. Sonett J. (2011a). Elevated plasma clara cell secretory protein concentration is associated with high-grade primary graft dysfunction. Am. J. Transpl. 11 (3), 561–567. 10.1111/j.1600-6143.2010.03431.x PMC3079443 21299834

[B28] Diamond J. M. Lederer D. J. Kawut S. M. Lee J. Ahya V. N. Bellamy S. (2011b). Elevated plasma long pentraxin-3 levels and primary graft dysfunction after lung transplantation for idiopathic pulmonary fibrosis. Am. J. Transpl. 11 (11), 2517–2522. 10.1111/j.1600-6143.2011.03702.x PMC3206646 21883907

[B29] Diamond J. M. Meyer N. J. Feng R. Rushefski M. Lederer D. J. Kawut S. M. (2012). Variation in Ptx3 is associated with primary graft dysfunction after lung transplantation. Am. J. Respir. Crit. Care Med. 186 (6), 546–552. 10.1164/rccm.201204-0692OC 22822025 PMC3480532

[B30] D'Ovidio F. Kaneda H. Chaparro C. Mura M. Lederer D. Di Angelo S. (2013). Pilot study exploring lung allograft surfactant protein a (Sp-a) expression in association with lung transplant outcome. Am. J. Transpl. 13 (10), 2722–2729. 10.1111/ajt.12407 24007361

[B31] Drummond R. A. Desai J. V. Hsu A. P. Oikonomou V. Vinh D. C. Acklin J. A. (2022). Human dectin-1 deficiency impairs macrophage-mediated defense against phaeohyphomycosis. J. Clin. Invest 132 (22), e159348. 10.1172/JCI159348 36377664 PMC9663159

[B32] Eklund L. Kangas J. Saharinen P. (2017). Angiopoietin-tie signalling in the cardiovascular and lymphatic systems. Clin. Sci. (Lond) 131 (1), 87–103. 10.1042/CS20160129 27941161 PMC5146956

[B33] Eva T. A. Barua N. Chowdhury M. M. Yeasmin S. Rakib A. Islam M. R. (2022). Perspectives on signaling for biological- and processed food-related advanced glycation end-products and its role in cancer progression. Crit. Rev. Food Sci. Nutr. 62 (10), 2655–2672. 10.1080/10408398.2020.1856771 33307763

[B34] Ferreira A. M. Rollins B. J. Faunce D. E. Burns A. L. Zhu X. Dipietro L. A. (2005). The effect of mcp-1 depletion on chemokine and chemokine-related gene expression: evidence for a complex network in acute inflammation. Cytokine 30 (2), 64–71. 10.1016/j.cyto.2004.12.006 15804597

[B35] Finetti F. Paradisi L. Bernardi C. Pannini M. Trabalzini L. (2023). Cooperation between prostaglandin E2 and epidermal growth factor receptor in cancer progression: a dual target for cancer therapy. Cancers (Basel) 15 (8), 2374. 10.3390/cancers15082374 37190301 PMC10136831

[B36] Finetti F. Travelli C. Ercoli J. Colombo G. Buoso E. Trabalzini L. (2020). Prostaglandin E2 and cancer: insight into tumor progression and immunity. Biol. (Basel) 9 (12), 434. 10.3390/biology9120434 PMC7760298 33271839

[B37] Frick A. E. Verleden S. E. Ordies S. Sacreas A. Vos R. Verleden G. M. (2020). Early protein expression profile in bronchoalveolar lavage fluid and clinical outcomes in primary graft dysfunction after lung transplantation. Eur. J. Cardiothorac. Surg. 58 (2), 379–388. 10.1093/ejcts/ezaa043 32267918

[B38] Greenland J. R. Guo R. Lee S. Tran L. Kapse B. Kukreja J. (2023). Short airway telomeres are associated with primary graft dysfunction and chronic lung allograft dysfunction. J. Heart Lung Transpl. 42 (12), 1700–1709. 10.1016/j.healun.2023.08.018 PMC10858720 37648073

[B39] Guzmán A. Hernández-Coronado C. G. Gutiérrez C. G. Rosales-Torres A. M. (2023). The vascular endothelial growth factor (vegf) system as a key regulator of ovarian follicle angiogenesis and growth. Mol. Reprod. Dev. 90 (4), 201–217. 10.1002/mrd.23683 36966489

[B40] Hagag A. A. Elmahdy H. S. Ezzat A. A. (2011). Prognostic value of plasma pro-adrenomedullin and antithrombin levels in neonatal sepsis. Indian Pediatr. 48 (6), 471–473. 10.1007/s13312-011-0074-1 21555796

[B41] Hamilton B. C. S. Kukreja J. Ware L. B. Matthay M. A. (2017). Protein biomarkers associated with primary graft dysfunction following lung transplantation. Am. J. Physiol. Lung Cell Mol. Physiol. 312 (4), L531-L541–L41. 10.1152/ajplung.00454.2016 28130262 PMC5407092

[B42] Hu Z. Chen H. Long Y. Li P. Gu Y. (2021). The main sources of circulating cell-free DNA: apoptosis, necrosis and active secretion. Crit. Rev. oncology/hematology 157, 103166. Epub 2020/12/01. 10.1016/j.critrevonc.2020.103166 33254039

[B43] Hunt M. L. Cantu E. (2023). Primary graft dysfunction after lung transplantation. Curr. Opin. Organ Transpl. 28 (3), 180–186. 10.1097/MOT.0000000000001065 PMC10214980 37053083

[B44] Ishiyama H. Tanaka T. Saito S. Koyama T. Kitamura A. Inoue M. (2023). Plasma mid-regional pro-adrenomedullin: a biomarker of the ischemic penumbra in hyperacute stroke. Brain Pathol. 33 (2), e13110. 10.1111/bpa.13110 35916272 PMC10041162

[B45] Iwata T. Philipovskiy A. Fisher A. J. Presson R. G. Chiyo M. Lee J. (2008). Anti-type V collagen humoral immunity in lung transplant primary graft dysfunction. J. Immunol. 181 (8), 5738–5747. 10.4049/jimmunol.181.8.5738 18832733 PMC2997998

[B46] Jin Y. Sun M. Lv X. Wang X. Jiang G. Chen C. (2020). Extracellular histones play a pathogenic role in primary graft dysfunction after human lung transplantation. RSC Adv. 10 (21), 12485–12491. 10.1039/d0ra00127a 35497627 PMC9051052

[B47] Ju C. Xu X. Zhang J. Chen A. Lian Q. Liu F. (2023). Application of plasma donor-derived cell free DNA for lung allograft rejection diagnosis in lung transplant recipients. BMC Pulm. Med. 23 (1), 37. 10.1186/s12890-022-02229-y 36703125 PMC9881379

[B48] Kanou T. Nakahira K. Choi A. M. Yeung J. C. Cypel M. Liu M. (2021). Cell-free DNA in human *ex vivo* lung perfusate as a potential biomarker to predict the risk of primary graft dysfunction in lung transplantation. J. Thorac. Cardiovasc Surg. 162 (2), 490–499.e2. 10.1016/j.jtcvs.2020.08.008 32928548

[B49] Keller M. Agbor-Enoh S. (2021). Donor-derived cell-free DNA for acute rejection monitoring in heart and lung transplantation. Curr. Transplant. Rep. 8 (4), 351–358. 10.1007/s40472-021-00349-8 34754720 PMC8570240

[B50] Keller M. Agbor-Enoh S. (2022). Cell-free DNA in lung transplantation: research tool or clinical workhorse? Curr. Opin. Organ Transpl. 27 (3), 177–183. Epub 2022/06/02. 10.1097/mot.0000000000000979 PMC9179944 35649108

[B51] Keller M. Bush E. Diamond J. M. Shah P. Matthew J. Brown A. W. (2021). Use of donor-derived-cell-free DNA as a marker of early allograft injury in primary graft dysfunction (pgd) to predict the risk of chronic lung allograft dysfunction (clad). J. Heart Lung Transpl. 40 (6), 488–493. 10.1016/j.healun.2021.02.008 PMC8169618 33814284

[B52] Keller M. Mutebi C. Shah P. Levine D. Aryal S. Iacono A. (2022b). Biological variation of donor-derived cell-free DNA in stable lung transplant recipients. J. Appl. laboratory Med. 7 (4), 901–909. Epub 2022/01/14. 10.1093/jalm/jfab171 35024828

[B53] Keller M. Sun J. Mutebi C. Shah P. Levine D. Aryal S. (2022a). Donor-derived cell-free DNA as a composite marker of acute lung allograft dysfunction in clinical care. J. Heart Lung Transpl. 41 (4), 458–466. Epub 2022/01/23. 10.1016/j.healun.2021.12.009 35063338

[B54] Keller M. B. Meda R. Fu S. Yu K. Jang M. K. Charya A. (2022c). Comparison of donor-derived cell-free DNA between single versus double lung transplant recipients. Am. J. Transpl. 22 (10), 2451–2457. Epub 2022/03/25. 10.1111/ajt.17039 PMC9508279 35322546

[B55] Keller M. B. Newman D. Alnababteh M. Ponor L. Shah P. Mathew J. (2024). Extreme elevations of donor-derived cell-free DNA increases the risk of chronic lung allograft dysfunction and death, even without clinical manifestations of disease. J. Heart Lung Transpl. 43 (9), 1374–1382. Epub 2024/05/06. 10.1016/j.healun.2024.04.064 PMC13186095 38705500

[B56] Képes Z. Dénes N. Kertész I. Hajdu I. Trencsényi G. (2023). Overview of prostaglandin E2 (Pge2)-Targeting radiolabelled imaging probes from preclinical perspective: lessons learned and road ahead. Int. J. Mol. Sci. 24 (8), 6942. 10.3390/ijms24086942 37108106 PMC10138785

[B57] Kim D. K. Kang S. H. Kim J. S. Kim Y. G. Lee Y. H. Lee D.-Y. (2023b). Clinical implications of circulating follistatin-like protein-1 in hemodialysis patients. Sci. Rep. 13 (1), 6637. 10.1038/s41598-023-33545-w 37095121 PMC10126138

[B58] Kim H. D. Bae H. Kang H. Lee H. Eum S. H. Yang C. W. (2024). Donor-derived cell-free DNA predicted allograft rejection and severe microvascular inflammation in kidney transplant recipients. Front. Immunol. 15, 1433918. Epub 2024/07/24. 10.3389/fimmu.2024.1433918 39044817 PMC11263016

[B59] Kim T. Kim S. J. Choi H. Shin T. R. Sim Y. S. (2023a). Diagnostic utility and tendency of bronchial and serum soluble receptor for advanced glycation endproducts (srage) in lung cancer. Cancers (Basel) 15 (10), 2819. 10.3390/cancers15102819 37345156 PMC10216359

[B60] King S. D. Chen S.-Y. (2020). Recent progress on surfactant protein A: cellular function in lung and kidney disease development. Am. J. Physiol. Cell Physiol. 319 (2), C316-C320–C20. 10.1152/ajpcell.00195.2020 32639871 PMC7500221

[B61] Krenn K. Klepetko W. Taghavi S. Lang G. Schneider B. Aharinejad S. (2007). Recipient vascular endothelial growth factor serum levels predict primary lung graft dysfunction. Am. J. Transpl. 7 (3), 700–706. 10.1111/j.1600-6143.2006.01673.x 17250560

[B62] Kuppusamy M. Ta H. Q. Davenport H. N. Bazaz A. Kulshrestha A. Daneva Z. (2023). Purinergic P2y2 receptor-induced activation of endothelial Trpv4 channels mediates lung ischemia-reperfusion injury. Sci. Signal 16 (808), eadg1553. 10.1126/scisignal.adg1553 37874885 PMC10683978

[B63] Lee J. C. Christie J. D. (2011). Primary graft dysfunction. Clin. Chest Med. 32 (2), 279–293. 10.1016/j.ccm.2011.02.007 21511090

[B64] Leon I. Vicente R. Moreno I. Ramos F. Solé A. Morales P. (2009). Plasma levels of N terminal pro-brain natriuretic peptide as a prognostic value in primary graft dysfunction and a predictor of mortality in the immediate postoperative period of lung transplantation. Transpl. Proc. 41 (6), 2216–2217. 10.1016/j.transproceed.2009.05.016 19715877

[B65] Li D. Weinkauf J. Hirji A. Nagendran J. Kapasi A. Lien D. (2021). Chest X-ray sizing for lung transplants reflects pulmonary diagnosis and body composition and is associated with primary graft dysfunction risk. Transplantation 105 (2), 382–389. 10.1097/TP.0000000000003238 32229774

[B66] Li Y. Liang B. (2023). Circulating donor-derived cell-free DNA as a marker for rejection after lung transplantation. Front. Immunol. 14, 1263389. Epub 2023/10/27. 10.3389/fimmu.2023.1263389 37885888 PMC10598712

[B67] Liang J. Cai Y. Shao Y. (2023). Comparison of presepsin and mid-regional pro-adrenomedullin in the diagnosis of sepsis or septic shock: a systematic review and meta-analysis. BMC Infect. Dis. 23 (1), 288. 10.1186/s12879-023-08262-4 37147598 PMC10160726

[B68] Lozano-Edo S. Sánchez-Lázaro I. Portolés M. Roselló-Lletí E. Tarazón E. Arnau-Vives M. A. (2022). Plasma levels of Serca2a as a noninvasive biomarker of primary graft dysfunction after heart transplantation. Transplantation 106 (4), 887–893. 10.1097/TP.0000000000003798 33901112

[B69] Magnusson J. M. Ricksten A. Dellgren G. Wasslavik C. Nordén R. Westin J. (2022). Cell-free DNA as a biomarker after lung transplantation: a proof-of-concept study. Immun. Inflamm. Dis. 10 (5), e620. Epub 2022/04/29. 10.1002/iid3.620 35478446 PMC9017613

[B70] Mak K. M. Png C. Y. M. Lee D. J. (2016). Type V collagen in Health, disease, and fibrosis. Anat. Rec. Hob. 299 (5), 613–629. 10.1002/ar.23330 26910848

[B71] Mattox A. K. Douville C. Wang Y. Popoli M. Ptak J. Silliman N. (2023). The origin of highly elevated cell-free DNA in healthy individuals and patients with pancreatic, colorectal, lung, or ovarian cancer. Cancer Discov. 13 (10), 2166–2179. Epub 2023/08/11. 10.1158/2159-8290.Cd-21-1252 37565753 PMC10592331

[B72] Nakata K. Okazaki M. Kawana S. Kubo Y. Shimizu D. Tanaka S. (2023). S100a8/A9 as a prognostic biomarker in lung transplantation. Clin. Transpl. 37 (9), e15006. 10.1111/ctr.15006 37115007

[B73] Nakhaei-Nejad M. Farhan M. Mojiri A. Jabbari H. Murray A. G. Jahroudi N. (2019). Regulation of von Willebrand factor gene in endothelial cells that are programmed to pluripotency and differentiated back to endothelial cells. Stem cells Dayt. Ohio 37 (4), 542–554. Epub 2019/01/27. 10.1002/stem.2978 30682218

[B74] Neves D. B. Rusi M. B. Diaz L. G. G. Salvalaggio P. (2016). Primary graft dysfunction of the liver: definitions, diagnostic criteria and risk factors. Einstein (Sao Paulo) 14 (4), 567–572. 10.1590/S1679-45082016RW3585 27783749

[B75] Nicolini G. Forini F. Kusmic C. Iervasi G. Balzan S. (2019). Angiopoietin 2 signal complexity in cardiovascular disease and cancer. Life Sci. 239, 117080. 10.1016/j.lfs.2019.117080 31756341

[B76] Ochoa A. E. Congel J. H. Corley J. M. Janssen W. J. Nick J. A. Malcolm K. C. (2023). Dectin-1-Independent macrophage phagocytosis of Mycobacterium abscessus. Int. J. Mol. Sci. 24 (13), 11062. 10.3390/ijms241311062 37446240 PMC10341562

[B77] Riera J. Senna A. Cubero M. Roman A. Rello J. Vall d'Hebron Lung Transplant Study Group I (2016). Primary graft dysfunction and mortality following lung transplantation: a role for proadrenomedullin plasma levels. Am. J. Transpl. 16 (2), 634–639. 10.1111/ajt.13478 26461449

[B78] Salama M. Andrukhova O. Hoda M. A. Taghavi S. Jaksch P. Heinze G. (2010). Concomitant endothelin-1 overexpression in lung transplant donors and recipients predicts primary graft dysfunction. Am. J. Transpl. 10 (3), 628–636. 10.1111/j.1600-6143.2009.02957.x 20055806

[B79] Sanchez-Gonzalez C. Fernández Aguilar J. L. Sánchez Pérez B. Muñoz M. Á. S. Daga J. A. P. Reyes M. P. (2022). Primary graft dysfunction: factor V's value for its early diagnosis. Transpl. Proc. 54 (9), 2531–2534. 10.1016/j.transproceed.2022.09.017 36273958

[B80] Santos J. Wang P. Shemesh A. Liu F. Tsao T. Aguilar O. A. (2023). Ccr5 drives nk cell-associated airway damage in pulmonary ischemia-reperfusion injury. JCI Insight 8 (21), e173716. 10.1172/jci.insight.173716 37788115 PMC10721259

[B81] Scholz A. Plate K. H. Reiss Y. (2015). Angiopoietin-2: a multifaceted cytokine that functions in both angiogenesis and inflammation. Ann. N. Y. Acad. Sci. 1347, 45–51. 10.1111/nyas.12726 25773744

[B82] Shah R. J. Diamond J. M. (2018). Primary graft dysfunction (pgd) following lung transplantation. Semin. Respir. Crit. Care Med. 39 (2), 148–154. 10.1055/s-0037-1615797 29590671

[B83] Shah R. J. Diamond J. M. Lederer D. J. Arcasoy S. M. Cantu E. M. Demissie E. J. (2012). Plasma monocyte chemotactic protein-1 levels at 24 hours are a biomarker of primary graft dysfunction after lung transplantation. Transl. Res. 160 (6), 435–442. 10.1016/j.trsl.2012.08.003 22989614 PMC3500407

[B84] Shah R. J. Wickersham N. Lederer D. J. Palmer S. M. Cantu E. Diamond J. M. (2014). Preoperative plasma club (clara) cell secretory protein levels are associated with primary graft dysfunction after lung transplantation. Am. J. Transpl. 14 (2), 446–452. 10.1111/ajt.12541 PMC3946770 24400993

[B85] Sherwood K. Weimer E. T. (2018). Characteristics, properties, and potential applications of circulating cell-free dna in clinical diagnostics: a focus on transplantation. J. Immunol. methods 463, 27–38. Epub 2018/09/30. 10.1016/j.jim.2018.09.011 30267663

[B86] Shi Y. Hu Y. Cui B. Zhuang S. Liu N. (2022). Vascular endothelial growth factor-mediated peritoneal neoangiogenesis in peritoneal dialysis. Perit. Dial. Int. 42 (1), 25–38. 10.1177/08968608211004683 33823711

[B87] Singh S. Anshita D. Ravichandiran V. (2021). Mcp-1: function, regulation, and involvement in disease. Int. Immunopharmacol. 101 (Pt B), 107598. 10.1016/j.intimp.2021.107598 34233864 PMC8135227

[B88] Singh V. Kaur R. Kumari P. Pasricha C. Singh R. (2023). Icam-1 and vcam-1: gatekeepers in various inflammatory and cardiovascular disorders. Clin. chimica acta; Int. J. Clin. Chem. 548, 117487. Epub 2023/07/14. 10.1016/j.cca.2023.117487 37442359

[B89] Somers J. Ruttens D. Verleden S. E. Vandermeulen E. Piloni D. Wauters E. (2015). Interleukin-17 receptor polymorphism predisposes to primary graft dysfunction after lung transplantation. J. Heart Lung Transpl. 34 (7), 941–949. 10.1016/j.healun.2015.03.009 25935436

[B90] Song S.-H. Kim K. L. Lee K.-A. Suh W. (2012). Tie1 regulates the Tie2 agonistic role of angiopoietin-2 in human lymphatic endothelial cells. Biochem. Biophysical Res. Commun. 419 (2), 281–286. 10.1016/j.bbrc.2012.02.009 22342979

[B91] Spahn J. H. Li W. Bribriesco A. C. Liu J. Shen H. Ibricevic A. (2015). Dap12 expression in lung macrophages mediates ischemia/reperfusion injury by promoting neutrophil extravasation. J. Immunol. 194 (8), 4039–4048. 10.4049/jimmunol.1401415 25762783 PMC4401083

[B92] Spoto S. Argemi J. Di Costanzo R. Gavira Gomez J. J. Salterain Gonzales N. Basili S. (2023). Mid-regional pro-adrenomedullin and N-terminal pro-B-type natriuretic peptide measurement: a multimarker approach to diagnosis and prognosis in acute heart failure. J. Pers. Med. 13 (7), 1155. 10.3390/jpm13071155 37511766 PMC10381388

[B93] Suzuki Y. Cantu E. Christie J. D. (2013). Primary graft dysfunction. Semin. Respir. Crit. Care Med. 34 (3), 305–319. 10.1055/s-0033-1348474 23821506 PMC3968688

[B94] Tiriveedhi V. Gautam B. Sarma N. J. Askar M. Budev M. Aloush A. (2013). Pre-transplant antibodies to Kα1 tubulin and collagen-V in lung transplantation: clinical correlations. J. Heart Lung Transpl. 32 (8), 807–814. 10.1016/j.healun.2013.06.003 PMC3763944 23856218

[B95] Valpione S. Gremel G. Mundra P. Middlehurst P. Galvani E. Girotti M. R. (2018). Plasma total cell-free DNA (cfdna) is a surrogate biomarker for tumour burden and a prognostic biomarker for survival in metastatic melanoma patients. Eur. J. cancer (Oxford, Engl. 1990) 88 (88), 1–9. Epub 2017/11/28. 10.1016/j.ejca.2017.10.029 PMC5769519 29175734

[B96] van de Stolpe A. van der Saag P. T. (1996). Intercellular adhesion molecule-1. J. Mol. Med. Berlin, Ger. 74 (1), 13–33. Epub 1996/01/01. 10.1007/bf00202069 8834767

[B97] Veraar C. Kirschner E. Schwarz S. Jaksch P. Hoetzenecker K. Tschernko E. (2022). Follistatin-like 1 and biomarkers of neutrophil activation are associated with poor short-term outcome after lung transplantation on va-ecmo. Biol. (Basel) 11 (10), 1475. 10.3390/biology11101475 PMC9598172 36290379

[B98] Weber J. Rajan S. Schremmer C. Chao Y.-K. Krasteva-Christ G. Kannler M. (2020). Trpv4 channels are essential for alveolar epithelial barrier function as protection from lung edema. JCI Insight 5 (20), e134464. 10.1172/jci.insight.134464 32931478 PMC7605532

[B99] Wu Y. Huang L. Li M. Cui X. Zhan Q. Wang C. (2023). The role of lung microbiota in primary graft dysfunction in lung transplant recipients. Clin. Transpl. 37 (12), e15152. 10.1111/ctr.15152 37788167

[B100] Xiang Y. Hwa J. (2016). Regulation of vwf expression, and secretion in Health and disease. Curr. Opin. Hematol. 23 (3), 288–293. Epub 2016/01/16. 10.1097/moh.0000000000000230 26771163 PMC5209751

[B101] Yang N. Wang M. Lin K. Wang M. Xu D. Han X. (2023). Dectin-1 deficiency alleviates diabetic cardiomyopathy by attenuating macrophage-mediated inflammatory response. Biochim. Biophys. Acta Mol. Basis Dis. 1869 (6), 166710. 10.1016/j.bbadis.2023.166710 37054997

[B102] Yang W. Cerier E. J. Núñez-Santana F. L. Wu Q. Yan Y. Kurihara C. (2022). Il-1β-Dependent extravasation of preexisting lung-restricted autoantibodies during lung transplantation activates complement and mediates primary graft dysfunction. J. Clin. Invest 132 (20), e157975. 10.1172/JCI157975 36250462 PMC9566897

[B103] Yoshimura T. (2018). The chemokine mcp-1 (Ccl2) in the host interaction with cancer: a foe or ally? Cell Mol. Immunol. 15 (4), 335–345. 10.1038/cmi.2017.135 29375123 PMC6052833

[B104] Young K. A. Dilling D. F. (2019). The future of lung transplantation. Chest 155 (3), 465–473. 10.1016/j.chest.2018.08.1036 30171860 PMC6435913

[B105] Yue Q. Song Y. Liu Z. Zhang L. Yang L. Li J. (2022). Receptor for advanced glycation end products (rage): a pivotal hub in immune diseases. Molecules 27 (15), 4922. 10.3390/molecules27154922 35956875 PMC9370360

[B106] Zaffiri L. Shah R. J. Stearman R. S. Rothhaar K. Emtiazjoo A. M. Yoshimoto M. (2019). Collagen type-V is a danger signal associated with primary graft dysfunction in lung transplantation. Transpl. Immunol. 56, 101224. 10.1016/j.trim.2019.101224 31325493

[B107] Zanetta L. Marcus S. G. Vasile J. Dobryansky M. Cohen H. Eng K. (2000). Expression of von Willebrand factor, an endothelial cell marker, is up-regulated by angiogenesis factors: a potential method for objective assessment of tumor angiogenesis. Int. J. cancer 85 (2), 281–288. Epub 2000/01/11. 10.1002/(sici)1097-0215(20000115)85:2<281::aid-ijc21>3.0.co;2-3 10629090

[B108] Zelniker T. A. Schwall D. Hamidi F. Steinbach S. Scheller P. Spaich S. (2023). Mid-regional pro-adrenomedullin and lactate levels for risk stratification in patients with out-of-Hospital cardiac arrest. Eur. Heart J. Acute Cardiovasc Care 12 (6), 364–371. 10.1093/ehjacc/zuad029 36943296 PMC10236520

[B109] Zhang W. Liu B. Jia D. Wang R. Cao H. Wu H. (2024). Application of graft-derived cell-free DNA for solid organ transplantation. Front. Immunol. 15, 1461480. Epub 2024/10/08. 10.3389/fimmu.2024.1461480 39376561 PMC11456428

[B110] Zhong W. Wang Q. Shen X. Du J. (2023). The emerging role of neutrophil extracellular traps in cancer: from lab to ward. Front. Oncol. 13, 1163802. 10.3389/fonc.2023.1163802 37188184 PMC10175598

[B111] Zou J. Duffy B. Slade M. Young A. L. Steward N. Hachem R. (2017). Rapid detection of donor cell free DNA in lung transplant recipients with rejections using donor-recipient hla mismatch. Hum. Immunol. 78 (4), 342–349. Epub 2017/03/08. 10.1016/j.humimm.2017.03.002 28267558 PMC5595356

